# Changes in platelet parameters in leukocytosis

**DOI:** 10.11604/pamj.2016.24.185.7510

**Published:** 2016-07-01

**Authors:** Nurinnisa Ozturk, Nurcan Kilic Baygutalp, Ebubekir Bakan, Gulsum Feyza Altas, Harun Polat, Emrullah Dorman

**Affiliations:** 1 Ataturk University Faculty of Medicine, Department of Medical Biochemistry, Erzurum, Turkey; 2 Dokuz Eylul University Faculty of Medicine, Department of Medical Biochemistry, Erzurum, Turkey

**Keywords:** Leukocytosis, platelet count, platelet parameters

## Abstract

**Introduction:**

In recent years, platelets are known to have a large variety of functions in many pathophysiological processes and their interaction with endothelial cells and leukocytes is known to play an important role in the pathophysiology of vascular inflammation. The aim of this study was to investigate the relationship between white blood cell count in conditions resulting in leukocytosis and platelet count and platelet parameters including mean platelet volume, platelet distribution width, and plateletcrit.

**Methods:**

White blood cell counts count and all platelet parameters were evaluated in 341 results of normal complete blood count (of which the white blood cell counts were within reference range, group 1) and 327 results of elevated white blood cell counts count (group 2).

**Results:**

There was a significant difference between these two groups in PLT counts and PCT values, being higher in Group 2. However, there was no statistically significant difference between two groups in MPV and PDW values. On the other hand, there were statistically significant, but weak, correlations between the WBC and platelet counts in both groups (p<0.01, r=0.235 for group 1, p<0.05, r=0.116 for group 2).

**Conclusion:**

As a conclusion PLT count and PCT values increase in infectious conditions. This study and previous studies show that PLTs are employed in infectious conditions but the exact mechanism and the exact clinical importance of this response remains to be cleared by further studies.

## Introduction

Platelets (PLTs) play an important role in the primary hemostasis and arterial thrombosis formation. They provide rapid protection against bleeding and catalyze the formation of stable blood clots [1, 2]. In addition to the fact that the central role of the platelets is to control bleeding and to induce thrombosis, they have different roles in inflammation, atherosclerosis, angiogenesis, antimicrobial host defense, and contribution to wound healing [1–4]. Activated PLTs can promote vascular inflammation, causing endothelial inflammation and subsequent leukocyte extravasation via their stored cytokines and chemokines [1, 4]. Over the last few years, several new and reportable parameters have been used in the routine complete blood count (CBC) analyzers [5]. Automated blood cell counters provide a platelet count and derive indices relating to the size of platelets. Size-related parameters are derived from the impedance platelet size distribution curve. Mean platelet volume (MPV) is calculated by dividing the plateletocrit (PCT) by the number of platelets, and therefore PCT is analogous to the red cell haematocrit. MPV is a potential marker of the platelet reactivity [6]. The platelet distribution width (PDW) is the width of the size distribution curve in femtoliter (fL) at the 20% level of the peak on the impedance platelet size distribution curve [5, 7]. Variation in platelet size is indicative of change in platelet function. Therefore platelet parameters are markers that are thought to be increased in response to systemic inflammation, and various studies have stated the relationship between these parameters and different inflammatory disease [7, 8]. Although high platelet count is not considered as an infection marker, some studies have suggested that infections may cause thrombocytosis [9, 10]. For this reason, we aimed in this study to investigate the relationship between leukocyte count (white blood cell count, WBC) in conditions resulting in leukocytosis and PLT count and its parameters, including MPV, PDW, and PCT.

## Methods

Ethical approval was granted by the Local Ethics Committee. We performed a retrospective study on the electronic data base of the laboratory information system between April 2014 and July 2014. The clinical and demographic records of the patients were also obtained from the hospital information system and the clinicians. CBC values were recorded, which were obtained by LH780 automated hematology analyzer (Beckman Coulter, Brea, CA) using VCS (volume- conductivity-light scatter) technology. The CBC analyses were carried out following daily quality control accomplish menton the basis of the manufacturer's instructions.341 subjects with normal WBC counts and the PLT parameters were included in the study as a control group (Group 1), and 327 subjects with elevated WBC count were evaluated for comparison with PLT counts and its parameters, which was called as Group 2. In the selection of the records from the database, some exclusion criteria were used, which included (a) patients with complicated infections or with very high-WBC count infections, (b) patients with infections secondary to solid organ or hematological malignancies, (c) patients with infections associated with solid organs or bone marrow transplantations, (d) patients with chronic infections, and (e) pediatric and octogenarian patients. The above-mentioned patients'results were not taken for evaluation. Statistical analyses were performed by statistical software program SPSS for Windows version 19.0 (SPSS, Chicago, IL, United States). Results were given as mean±standard deviation (SD) and minimum and maximum (min-max) values. Since the variables did not show normal distribution by Kolmogorov-Smirnov test, a nonparametric Mann-Whitney Test U was performed. The correlation between WBC and PLT parameters were assessed using Spearman correlation analyses.

## Results

**Figure 1:**
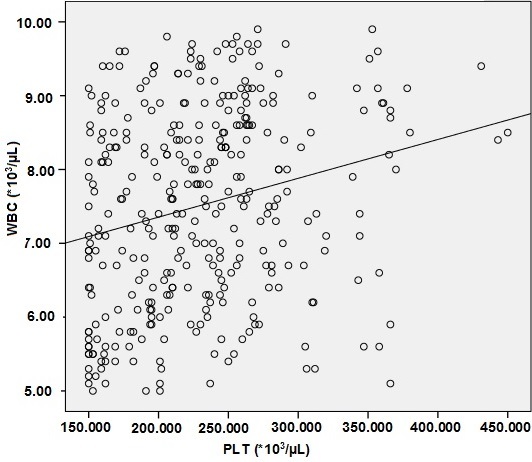
The scatter plot of PLT counts and WBC counts in group 1 (WBC counts within reference range) (p=0.000, r^2^=0.055)

**Figure 2:**
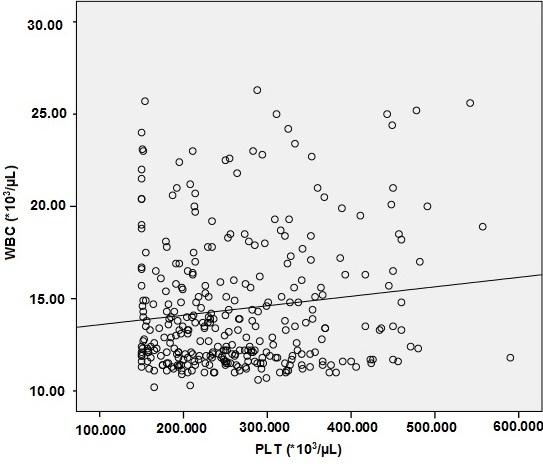
The scatter plot of PLT counts and WBC counts in group 2 (WBC counts higher than upper limit of reference range) (p=0.019, r=0.013)

We reviewed records of 668 CBC results. The mean age of subjects was 46.9 ± 18.3 years and range 18-75 years for group 1 and 47.3 ± 19.1 years and range 18-75 years for group 2. There were not statistically significant differences between two groups with respect to age. The statistical evaluation of WBC, PLT, MPV, PDW, and PCT values of the two groups is given in Table 1. There was a significant difference between these two groups in PLT countsand PCT values (p < 0.05), being higher in Group 2. However, there was no statistically significant difference between two groups in MPV and PDW values (p>0.05). On the other hand, there were statistically significant, but weak, correlations between the WBC and platelet counts in both groups (p < 0.01, r = 0.235 for group 1, p<0.05, r = 0.129 for group 2) ( Figure 1, Figure 2).

**Table 1:** WBC, PLT, MPV, PDW, and PCT values in two groups

Parameters	Group 1 (n=341)		Group 2 (n=327)		p and z values
	Mean±SD	Min-Max	Mean±SD	Min-Max	
WBC(*10^3^/µL)	7.51±1.32	5.00-9.90	14.19±2.89	11.00-20.00	p=0.000* z=-22.364
PLT(*10^3^/µL)	230±59.1	150-450	263±89.6	150-590	p=0.000* z=-4.243
**MPV(fL)**	8.26±0.93	6.60-10.90	8.22±0.97	6.60-10.90	p=0.444 z=-0.766
**PCT (%)**	0.18±0.04	0.10-0.45	0.21±0.07	0.10-0.54	p=0.000* z=-4.586
**PDW (%)**	16.84±0.69	14.60-19.70	16.89±0.71	14.90-19.70	p=0.603 z=-0.520

WBC: white blood cell count, PLT: platelet count, MPV: mean platelet volume, PDW: platelet distribution width, PCT: plateletocrit count, p:test statistic p value, z:, test statistic z value, SD: Standart deviation

* Statistically significant p value

## Discussion

This study was aimed to demonstrate that PLT counts and PLT parameters can probably be used as adjunct to clinical evaluation of infectious circumstances. PLTs have not only hemostatic functions but also are considered as inflammatory anucleate cells because many studies demonstrated that PLT counts and PLT parameters are strongly associated with inflammatory and infectious conditions [1, 2, 11, 12]. In the present study, we found that the PLT counts and PCT values significantly increased in Group 2 when compared with normal WBC count Group 1. In addition, we have found that MPV and PDW values were not significantly different in both groups. PCT, MPV and PDW values in CBC results are not usually considered in daily practise by clinicians. However, there are some recently-published studies evaluating the relationship between these parameters and various conditions, including coronary artery disease [6], endometriosis [13], cerebral infarction [14] diabetes mellitus [15, 16], pulmonary tuberculosis [17, 18], and inflammation [8, 12].

Our results showed that PLT counts were increased in infectious circumstances while MPV did not change. However, Zareifar et al. [12] reported in their study that PLT counts also increased in infectious and inflammatory diseases, as in our study, but MPV decreased, proposing that platelet parameters can be considered as reliable markers for assessment of disease activity. In a study, it is revealed that the PCT and PDW values were elevated in patients with tuberculosis, a chronic infection [17]. We however, selected the patients with acute infection in general and found that PCT values significantly increased and PDW values did not change. We tried to demonstrate the relationship between the platelet indices and leukocytosis. In our study, the patients (Group 2) had simple infections being able to be treated with simple methods. However such a relationship was also presented in severe infections associated with intensive care unit [7]. Zhang et al. [7] investigated this association between PLT indices and mortality of critical-ill patients and reported that this relationship was due to the inflammation.

These findings show that a linkage of PLT indices is present not only in simple but also in severe infections, and these indices can thus be used for daily clinical practise. In any simple and severe infectious circumstance, the decision for clinical treatment depends on a variety of clinical and laboratory data, and each data can be used for management of this infectious state and prediction of the prognosis. There is a well-known close relationship between leukocytes and platelets especially in inflamed endothelium [4, 19]. Leukocytes can roll on a template of adherent platelets, firmly adhere, and then transmigrate through the adherent platelets [19]. It is thought that PLTs are one of the first responding anucleate cells during the development of sepsis. Platelet activation readouts have been suggested as biomarkers for the development of septic complications and have been related to prognosis [20]. Although the changes in PLT count are common during sepsis [21], the relation of leucocytes to platelets is not clear in simple bacterial and viral infections. In addition, it has been reported that leukocytosis is a risk factor for thrombosis in polycythemia vera and essential thrombocythemia [22, 23], and leukocytosis is an independent, strong risk factor predicting major vascular events in essential thrombocythemia, particularly in the category of the younger and asymptomatic patients [22].

## Conclusion

PLT count and PCT values increase in infectious conditions. This study and previous studies show that PLTs are employed in infectious conditions but the exact mechanism and the exact clinical importance of this response remains to be cleared by further studies. Our study has a limitation that number of patients and controls were small.

### What is known about this topic

- MPV is usually increased in infectious conditions;
- PLT and PCT count are not usually altered in infectious conditions.

### What this study adds

- PLT count is increased in leukocytosis caused by infectious conditions;
- MPV isn’t changed in leukocytosis caused by infectious conditions.
